# Report on Cow-Pox

**Published:** 1873-06-01

**Authors:** J. E. Thayer

**Affiliations:** Menasha, Wis.


### REPORT ON COW-POX.

BY J. E. THAYER, M.D., MENASHA, WIS.

By order of the Board of Supervisors of the town of Metomen, Fond du Lac Co., Wis., December 1st, 1871, I was directed to vaccinate and revaccinate the inhabitants of said town.

Acting upon the oft-repeated popular opinion that cow-pox is modified small-pox, I determined on instituting a series of experiments that might result in the development of light touching this subject, upon which a conflicting medical opinion has been rampant since the days of Jenner. Many there are that contend that the primary cow-pox virus is obtained from the cow in a mysterious way. Others assert that the insertion of variolous virus into the cow is subjecting a community to the devastating influence of small-pox ; and thus the wail of horror goes up, mingled with groans of the adherents of the bougency stock.

I have investigated this subject for a year past, and have called to my assistance physicians of undoubted integrity, who have witnessed the passage from time to time of the first transmission from the cow of variolous inoculation through the unprotected infant; and the result of my wearisome investigation has led me decidedly in favor of the primary cow-pox being obtained by true variolous inoculation or contact of the bovine tribe with true small-pox, and not from varioloid; failing as I have, in every instance, to transmit the latter disease through the cow. That the cow is susceptible to the contagion of small-pox will hereinafter appear; and in its passage through that animal it is so modified that it becomes non-contagious from one animal to another, consequently resulting in its protective power to the human species.

Not having the acuteness of vision that characterizes the Poughkeepsie Seer, I have failed to see that the humanized virus may not be materially improved by its passage through the cow, and that its continued transmission through the human subject will result in debility of the virus, consequently becoming impregnated with diseases latent in the human system.

Fearing that my remarks may become tedious, I proceed to state that, in the outset of my investigations, I addressed a letter to Prof. E. Andrews, of Chicago, asking him if the primary cow-pox was obtained by the passage of the variolous virus through the cow, to which he replied : "If so obtained, in its passage through the human subject, the constitutional symptoms would be much more severe; but it is a point not yet settled by the profession."

Notwithstanding the adverse opinion and experiments of Ephraim Cutter, M.D., of Wobum, Mass., November 26th, 1859, I proceeded, on the 15th of December, 1871, assisted by P. W. Dingman, Treasurer of said town of Metomen, to insert the same variola virus, obtained through the kindness of Dr. Rogers, of the city of Ripon, into one of three milch cows, stall-fed, standing side by side, and owned by myself, by lifting up a portion of thin skin in four places on the bag and two near the vulva, and passing a bistoury through; and after the flow of blood had ceased, I throughly lodged the half of a variolous scab within the punctured surface.

On the seventh day after inoculation the pulse rose 30 beats above the normal standard, with an indisposition to receive food, but gradually improving in this respect, when, on the fourteenth day, appeared as well as usual, with twenty-six pustules occupying the bag, nose, eyelids, and vulva, well filled with lymph, drying down gradually, with a depression in the center, and scaling off from the twenty-first to the twenty-fifth day.

Case II.—I inoculated another cow with virus obtained from the first animal, with about the same constitutional symptoms as the first, producing a virus that worked in the human subject more agreeably than the first.

Case III.—I obtained a scab of humanized virus from Prof. E. Andrews at the time of the interrogation, a part of which I carried through the system of a calf, producing a virus that worked in the human system with more vigor and certainty than the remainder of the same scab.

Case IV.—I placed several scabs from a purely variolous subject upon a piece of smooth siding, under the nose of a young heifer, applying heat underneath until the fumes thoroughly permeated the nostrils, which produced in the animal the same disease, with corresponding constitutional results as appeared in the first cow inoculated, which virus was used by myself and others for the vaccination of the human subject.

CASE V.—The introduction of equal parts of cow-pox virus and small-pox virus produced about the same symptoms in the animal, and resulted in the production of about the same amount of virus, which worked as agreeably in the human system as any of the above forms.

My investigations have continued from time to time for the period of one year, using in that time twenty-one animals. And in support of the foregoing investigations, and consequent views thereof, I cite the readers of THE EXAMINER to the following authorities:

*First.*—Wood's Practice of Medicine, 1st volume, page 406.

*Second.*—To a report of a committee appointed by the American Medical Association, in which they state: "Knowing as we do that small-pox and cow-pox are in reality the same disease, the latter being merely deprived of its virulence by having previously passed through the system of the cow."—*Med. Examiner for April,* 1865.

*Third.*—A paper read by Mr. Seanoix before the Academy of Medicine, Paris, in which he says: "The revaccination with animal matter succeeds more frequently than with matter obtained from human beings; that vaccinations with heifer matter is extremely easy; and that such inoculations are highly useful in epidemics of small-pox, as large supplies of vaccine matter may rapidly be sent to extensive tracts of country."—*Lon. Lancet.*

*Fourth.*—"By many persons cow-pox is believed to be nothing more than a modification of small-pox, which is a disease that will certainly affect the cow. Cloths have been taken from patients laboring under small-pox and laid on cows, and they have had the disease called cow-pox."—*Study of Medicine, by John Mason Good, page* 617.

In connection with the foregoing authorities I might refer to Drs. Elliotson and Thompson, of the Royal Public Dispensary of Edinburgh, or Woodville of the Small-Pox Hospital of London; but enough has been said touching this point.

EFFECTS OF COW-POX RESULTING FROM VARIOLOUS TRANSMISSION UPON THE HUMAN SYSTEM.

On the fourteenth day of the disease of the first cow inoculated, I vaccinated my child, then two years old. The constitutional symptoms were much more severe than those resulting from humanized virus. From the eighth to the twelfth day sixteen pustules made their appearance, which filled and passed through the regular stages in a highly satisfactory manner. During the disease in my child I induced the presence of four children about the same age, at different times, and brought them in close proximity, to detemine its contagion; and after becoming fully satisfied of the non-contagion, on the twentieth day I vaccinated the four children with the virus obtained from my child.

Dr. Rogers, of the city of Ripon, visited my child, and several other cases, at different times, for the purpose of arriving at a just conclusion, and after becoming so convinced, used the virus in his practice with good satisfaction. I furnished quite a number of physicians with the virus thus originating, with perfect satisfaction to themselves and patients. Drs. Searles and Frost, of the city of Wausan, an extensive lumbering district, were then engaged in a wide-spread epidemic of small-pox, where some forty cases existed, and the spread of which was controlled to their entire satisfaction by the use of this matter.

I vaccinated, under the order of the Board of Supervisors, 957 persons, with gratifying results, out of which number were 406 revaccinations, the latter passing through the several stages with about the same severity as humanized virus does with the unprotected.

*Method of Insertion.—First,* By simple scarification in three different places on each arm, and applying the virus previously moistened with water, on glass.

*Second,* By saturating a thread in the moistened virus, and, with a surgeon's needle, drawing it under the skin in three or four places.

*Third,* By the use of the impregnated quill; but my investigations led me to favor the scarification process.

*Mode of Preserving.*—Acting under the suggestion of Prof. E. Andrews, in an article of some years since, I immersed the scabs in pure glycerine, well corked, and kept in a cool place, using the last about a month since, fourteen months after they were taken from the cows, with as active and satisfactory results as when first obtained.

Another fact in connection with my investigations worthy of note, was the existence of a wide-spread epidemic of scarlatina; and, upon the appearance of the vaccine disease, this epidemic rapidly disappeared. I was called at several different times to visit cases of scarlatina in private families of five or six children, unprotected, and vaccinating the remaining ones, without a single attack of the fever in those vaccinated. In three cases I vaccinated, while under the premonitory symptoms, and the disease passed through its regular stages quite mildly, when on the fourteenth day the vaccine disease sprang up from its dormant condition, without any previous symptoms, and worked very kindly.

How far, and to what extent vaccination will protect the system against this disease I am unable to say.

